# Hypophosphatemic osteomalacia secondary to jaw phosphaturic mesenchymal tumor: a case report with literature review

**DOI:** 10.3389/fsurg.2026.1903638

**Published:** 2026-08-26

**Authors:** Wuxiao Cui, Ting Liu, Shuqian Mo, Chaofeng Yang, Wei Deng

**Affiliations:** 1 School of Stomatology, Hainan Medical University, Haikou, China; 2 Department of Oral and Maxillofacial Surgery, Hainan Affiliated Hospital of Hainan Medical University, Hainan General Hospital, Haikou, China

**Keywords:** ^68^Ga-DOTA-TATE-PET/CT, case report, hypophosphatemic osteomalacia, jaw, paraneoplastic syndrome, phosphaturic mesenchymal tumors, tumor-induced osteomalacia

## Abstract

Tumor-induced osteomalacia (TIO) is a rare paraneoplastic syndrome characterized by renal phosphate wasting, resulting in hypophosphatemia and altered bone turnover. It is most frequently caused by phosphaturic mesenchymal tumors (PMTs), a rare and distinct group of neoplasms. Patients with TIO exhibit clinical features of osteomalacia, including bone and muscle pain, severe muscle weakness, gait disturbance, and increased susceptibility to fracture. PMTs exhibit pananatomic distribution, in soft tissue or bones, but are most commonly found in the lower extremities, followed by the head and neck. Most head and neck tumors are located in the sinus area, while jaw involvement is exceptionally rare. Diagnosis is difficult and the misdiagnosis rate is high. A case PMT occurring in the jaws are presented to highlight diagnostic features and challenges. Notably, PMTs are benign tumors and their removal results in correction of the biochemical aberrations that underly the TIO.

## Introduction

Tumor-induced osteomalacia (TIO) is an acquired hypophosphatemic osteomalacia resulting from excessive secretion of fibroblast growth factor 23 (FGF23) by a tumor, which leads to reduced renal phosphate reabsorption and increased urinary phosphate excretion. It is most frequently caused by phosphaturic mesenchymal tumors (PMTs), a rare benign mesenchymal neoplasm characterized by excessive FGF23 production. Clinically, patients present with weakness, bone pain, skeletal deformities, and multiple fractures. Following tumor resection, serum FGF23 levels decrease, serum phosphorus levels rise, and the patient's clinical condition improves. PMTs most commonly arise in the extremities; jaw involvement is rare. Therefore, we present a case of tumor-induced osteomalacia caused by a phosphaturic mesenchymal tumor located in the jaw, along with a review of the literature.

## Case presentation

A 46-year-old male patient developed rib and back pain 9 years ago. He was admitted to Hainan Orthopaedic Hospital in 2017, and diagnosed with osteoporosis despite treatment with calcium supplements, his symptoms did not significantly respond.

**Figure 1:**
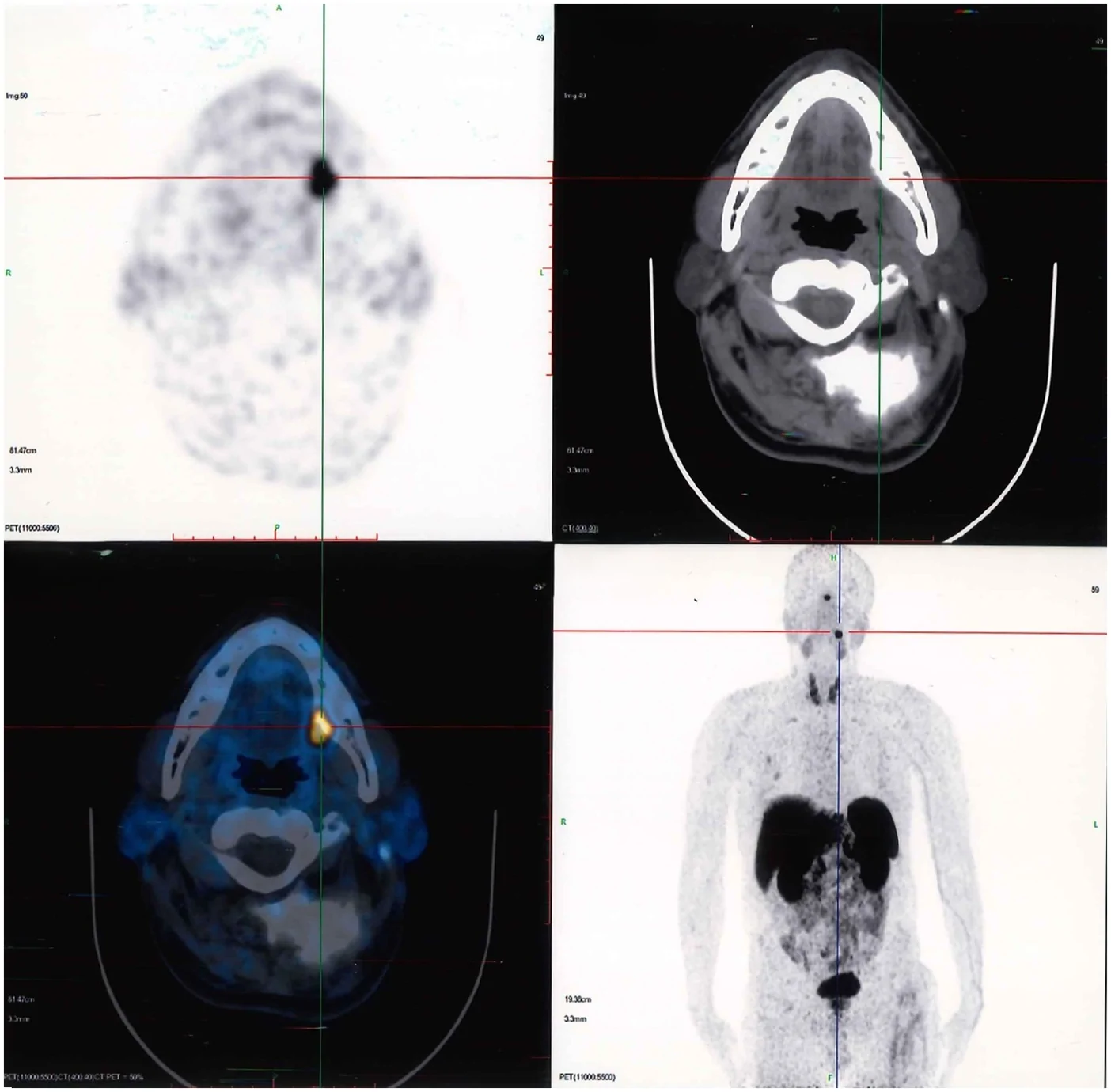
^68^Ga-DOTATATE PET/CT showed focal radiotracer uptake in the left mandibular lesion (lesion SUVmax = 10.3), accompanied by a low-density nodular lesion in the bone tissue and localized enlargement in the medial region of the left molar bone.

**Figure 2:**
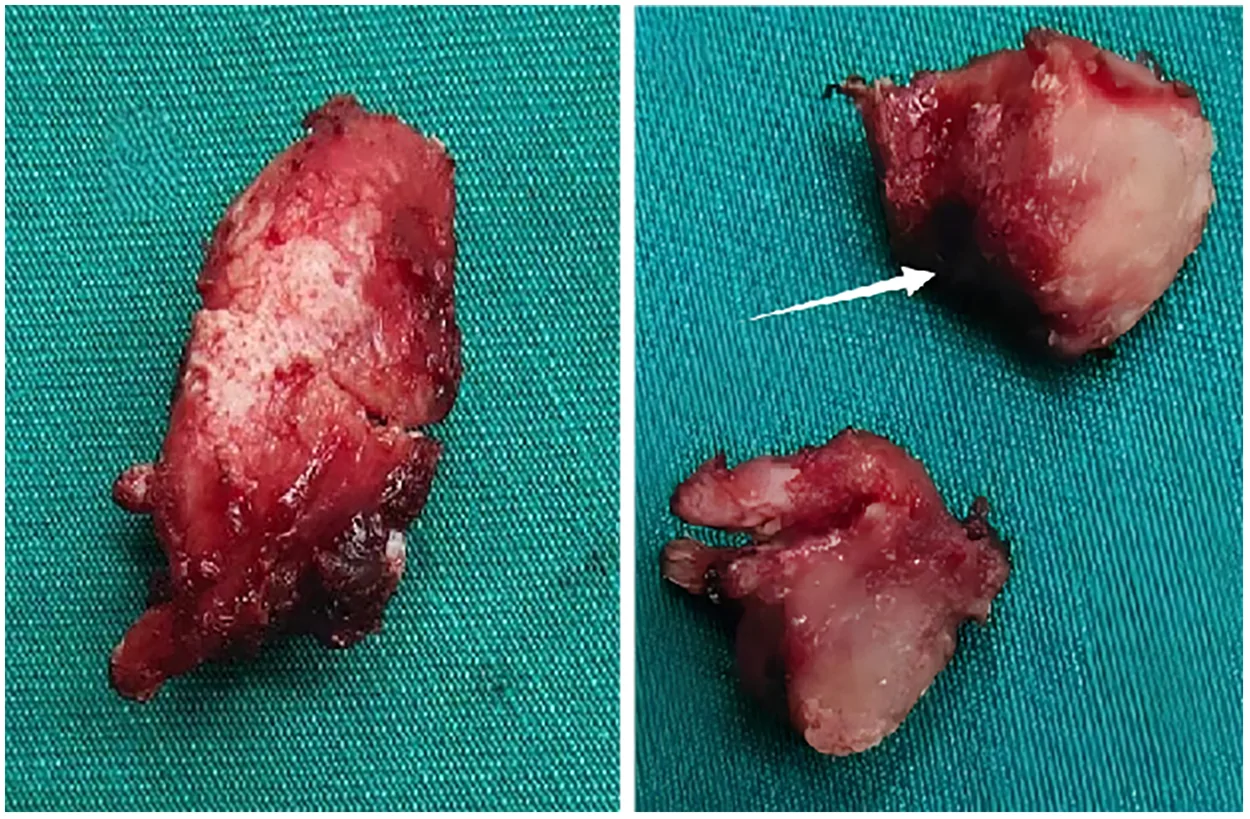
Left mandibular mass: the lesion measured 3 × 1 × 1 cm. Upon incision, the mass appeared grayish-brown and grayish-white.

**Figure 3:**
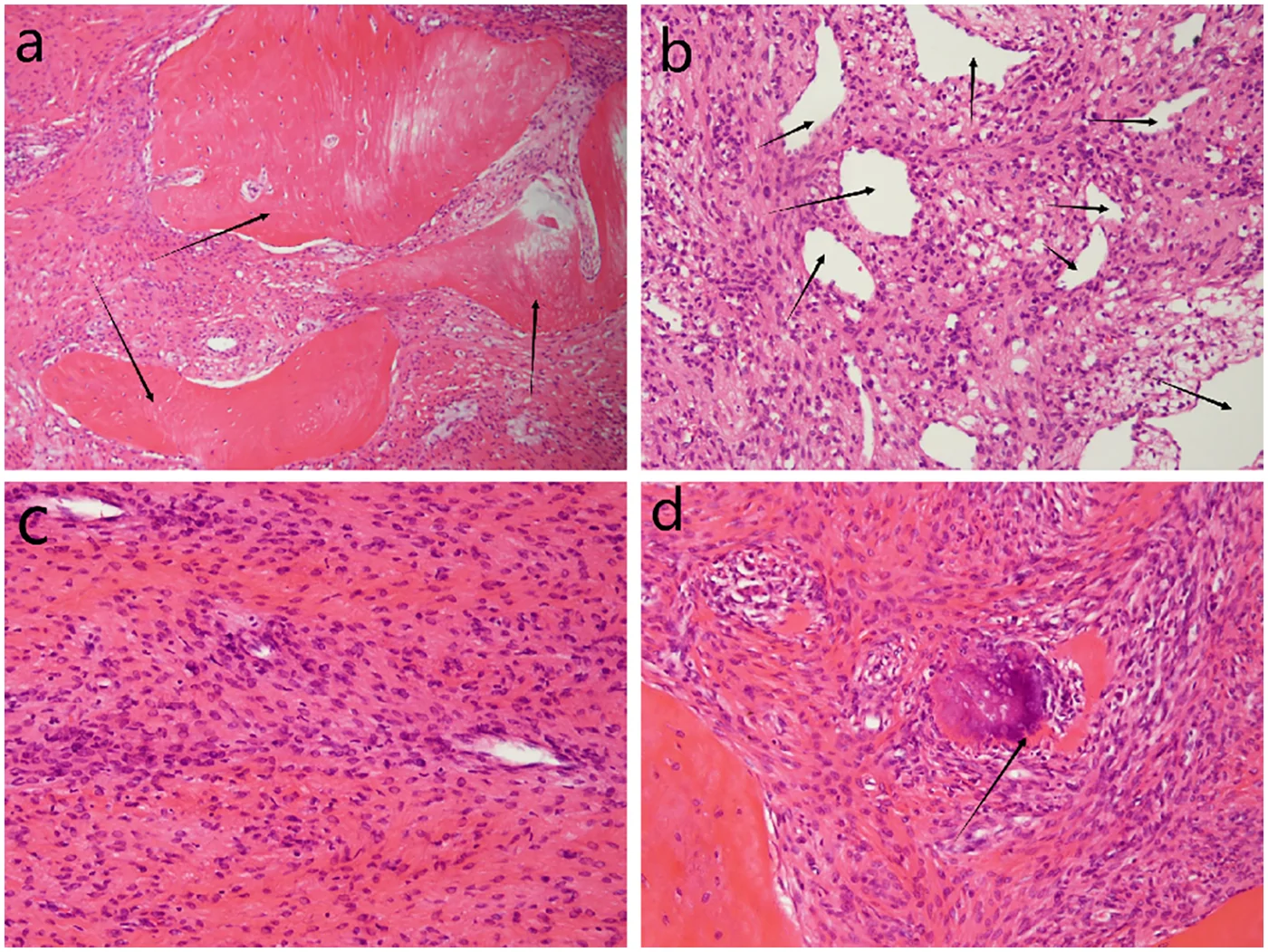
**(a)** the tumor's calcified tissue (HE, 10 × 20). **(b)** Numerous vascular structures are present in the tumor, such as capillary networks, sinusoids, thick-walled, malformed vessels, staghorn-like vessels and larger thin-walled vessels (HE, 10 × 20). **(c)** areas with loose spindle cells (HE, 10 × 20). **(d)** Smudgy calcification with irregular calcified nodules is present within the tumor (HE, 10 × 20).

In 2020, the patient developed lower limb joint pain and muscle weakness and experienced difficulty climbing stairs. The patient had previously been hospitalized in the Department of Endocrinology because of generalized joint pain. During hospitalization, serum calcium levels were within the normal range. The patient received calcitriol (0.25 μg once daily) and calcium carbonate (0.75 g twice daily) as anti-osteoporosis therapy for approximately two weeks. Serum calcium levels were monitored repeatedly during treatment and remained within the normal range. Importantly, no phosphate supplementation was administered before surgery. Despite treatment with calcitriol and calcium carbonate, repeated measurements showed persistent hypophosphatemia without substantial improvement. After a medical examination, he was determined to have hypophosphatemic osteomalacia. ^18^F-FDG PET/CT imaging revealed a slight increase in focal FDG metabolism in the anterior tissue space of the right knee joint, accounting for inflammation, but no obvious abnormalities were found. Knee surgery was performed but there was no significant improvement after knee joint surgery. In November 2021, the patient experienced a fall at home, resulting in a fracture of the left femoral head, and underwent fracture reduction fixation at Qionghai People's Hospital. Due to chronic, persistent pain, a ^68^Ga-DOTA-TATE-PET/CT examination revealed a mass in the left mandible (Figure 1). Based on radionucleotide imaging, this finding was considered highly suggestive of a PMT. Subsequent head and neck examination revealed a mass in the left mandible with an unclear boundary, hard texture, and no tenderness, extending from the region of the mandibular third molar to the second molar. Laboratory test results indicated a serum phosphorus level of 0.53 mmol/L [Reference ranges (RR):0.85–1.51 mmol/L]. When the blood phosphorus level is less than 0.53 mmol/L, the urine phosphorus should be close to 0. However, the patient's two urine phosphorus levels were 5.82 mmol/L and 4.50 mmol/L respectively, suggesting reduced phosphorus reabsorption, which supports the manifestation of hypophosphatemic osteomalacia. Serum calcium level of 2.23 mmol/L (RR: 2.11- 2.52 mmol/L), parathyroid hormone level of 59.5pg/mL (RR: 15.0–68.3pg/mL), 1,25-dihydroxyvitamin D level of 38.3 ng/mL (RR: ＞30 ng/mL) and alkaline phosphatase level of 151.8U/L (RR: 45-125U/L). He was Human leukocyte antigen B27 (HLA B27) negative. Based on the patient's symptoms, ^68^Ga-DOTA-TATE-PET/CT results, and biochemical examination, the preliminary diagnosis: Left mandibular mass consistent with PMT. Surgical treatment was performed. Intraoperatively, the tumor mass was grayish-brown in color, about 3.0 × 1.0 × 1.0 cm in size, and unencapsulated (Figure 2). The tumor was completely resected with an approximately 5-mm macroscopic surgical margin. The surgical wound defect was packed with a gelatin sponge, and it was sutured. After surgery, histopathologic examination revealed features consistent with PMT (Figure 3). Histopathological examination of the resected lesion revealed abundant vascular structures, including capillary networks, sinusoids, thick-walled malformed vessels, staghorn-like vessels, and larger thin-walled vessels, accompanied by loosely arranged spindle-shaped mesenchymal cells and characteristic smoky/grungy calcification with irregular calcified deposits. Based on these morphological findings, together with the clinical and imaging findings, the pathological diagnosis was phosphaturic mesenchymal tumor. Immunohistochemical staining for PMT-associated markers, including FGF23, SSTR2A, and CD56, was not performed, which represents a limitation of this report. One month after surgery, the patient's serum phosphorus level was 1.96 mmol/L (RR: 0.85–1.51 mmol/L) and serum calcium was 2.28 mmol/L (RR: 2.11–2.52 mmol/L). Although FGF23 and TmP/GFR measurements were unavailable, the diagnosis was supported by clinical, biochemical, imaging, pathological, and postoperative responses. At 2 months of follow-up, the patient's lumbosacral and limb pain was completely relieved, he moved freely, and his blood phosphorus level remained with normal range. At 2 years of follow-up, the patient could walk with a brisk gait and his blood phosphorus level was normal.

A literature search was performed in PubMed, Web of Science, and Embase databases from inception to today using the following keywords: (“phosphaturic mesenchymal tumor” OR “tumor-induced osteomalacia”) AND (“jaw” OR “mandible” OR “maxilla”). Reports describing PMTs involving the jaw region with available clinical information were included. Data regarding tumor location, symptoms, biochemical findings, imaging modalities, treatment, and outcomes were extracted. Excluding some cases where certain locations are not in the jawbone or specific conditions could not be confirmed, and excluding repeated cases, the results are presented in Table 1.

**Table 1:** Summary of reported phosphaturic mesenchymal tumors involving the jaw region.

Reference	No. of cases	Tumor location	Biochemical findings	Clinical characteristics	Imaging	Treatment modality	Outcome	Duration of follow-up	Recurrence status
Nitzan DW et al. (1) (1981)	1	mandibular	NM	muscular weakness and aching	x-ray	surgical resection	substantial improvement	NM	NM
J L Dupond et al. (2) (2005)	1	mandibular	extreme hypo phosphatemia with normal calcium level and an increase in total alkaline phosphatase et al	multiples fractures	^18^F-FDG PET/CT SCAN	surgical resection	relieved all symptoms	NM	NM
Victoria L Woo et al. (3) (2009)	1	mandibular	hypophosphatemia	back and flank pain	CBCT/i-CAT Classic CBCT	conservative curettage	substantial improvement	4 months	NM
Yoshiyuki Mori et al. (4) (2010)	1	maxilla	high serum alkaline phosphatase; hypophosphatemia; elevated FGF 23	pain in the joint and difficulty in walking	CT and MRI	surgical resection	greatly improved	15 months	NM
Lisa Luo et al. (5) (2013)	1	mandibular	calcium and phosphorous levels were normal	NM	US/CT/ PET-CT	manage her conservatively	NM	4 months	NM
V. Monappa et al. (6) (2014)	1	mandibular	low to normal serum calciu, low serum phosphorus and elevated serum alkaline phosphatase	multiple bone pains, generalized muscle aching and pathological fracture	CT	surgical resection	symptoms improved	1 year	no evidence of tumour recurrence
Elisa Fernández-Cooke, MD et al. (7) (2015)	1	mandibular	hypophosphatemia; elevated FGF 23	limp	MRI and octreotide scan	percutaneous computed tomography–guided radiofrequency, local instillation of triamcinolone, and oral propranolol	improved	4 years	NM
O Emodi et al. (8) (2018)	1	maxilla	low 1,25-dihydroxy vitamin D and hypophosphatemia, suspicious for elevated FGF23	pain, weakness, and spontaneous multiple bone fractures, loss of ambulatory ability	octreotide scan, a ^68^Ga-DOTA-TATE-PET/CT et al.	surgical resection	complete reversal of the patient's health condition	NM	NM
Huanwen Wu et al. (9) (2019)	22	maxilla/ mandibular	hypophosphatemia	bone pain and muscle weakness	octreotide scan, a ^68^Ga-DOTA-TATE-PET/CT et al.	21 patients with complete resection	no evidence of disease and normal phosphatemia at last follow-up	17 had follow-up information of more than 2 months	All 17 patients were alive with no evidence of disease and normal phosphatemia at last follow-up
Jinson Paul et al. (10) (2019)	1	mandibular	hypophosphatemia; elevated FGF 23	bone pains and muscle weakness	^68^Ga-DOTA-TATE-PET/CT	surgical resection	completely asymptomatic	1 year	NM
Rishabh P Acharya et al. (11) (2019)	1	mandibular	hypophosphatemia, low serum 1,25 dihydroxy vitamin D_3_, and elevated FGF23	bone pains	PET/CT CBCT	surgical resection	pain had markedly decreased	131 days	no evidence of residual or recurrent tumor
Sunil Kumar Mishra et al. (12) (2019)	1	mandibular	hypophosphatemia, decreased %TRP, and inappropriately normal serum FGF-23 level	bone pains and muscle weakness	^68^Ga-DOTA-TATE-PET/CT	RFA	remarkable clinical improvement	10 months	NM
Ravikumar Shah et al. (13) (2019)	2	maxilla	hypophosphatemia; elevated FGF 23	bone pains and muscle weakness	^68^Ga-DOTA-TATE-PET/CT	surgical resection	improved	12/48 months	NM
Dongmei Li et al. (14) (2020)	17	maxilla/ mandibular	hypophosphatemia, phosphaturia, and abnormal 1, 25-dihydroxy vitamin D	bone pain and muscle weakness	octreotide scan, a ^68^Ga-DOTA-TATE-PET/CT et al.	surgical resection	no evidence of disease and normal phosphatemia at last follow-up	4 months to 22 years	Two patientshad repeated recurrences
Eeva M Ryhänen et al. (15) (2021)	1	mandibular	Hypophosphatemia; high serum FGF23-concentration	bone pain and muscle weakness	CT	surgical resection	improved	2 years	NM
Zhenzhen Zhu et al. (16) (2021)	1	mandibular	NM	bone pain and muscle weakness	a ^68^Ga-DOTA-TATE-PET/CT, ^99^ Tc ^m^ -OCT	Mandible tumor resection	improved	5 months	no evidence of disease
Deepika Mishra et al. (17) (2021)	1	mandibular	raised serum alkaline phosphate; low serum phosphate and 1,25 (OH)_2_ D_3_levels; elevated FGF 23	bone pains and muscle weakness	CECT, MRI, PET/CT with 18 fluorodeoxyglucose (FDG)	surgical resection	dramatically resolved	NM	NM
Anna Herden et al. (18) (2022)	1	maxilla	NM	bone pains and fracture	^68^Ga-DOTA-TATE-PET/CT	the partial resection of the Maxilla	the level of FGF-23 in blood decreased	NM	NM
Ha Nguyen Thi et al. (19) (2023)	1	maxilla	low serum ionized calcium; hypophosphatemia; elevated alkaline phosphatase; low 1,25(OH)2D; Serum FGF23 level	bone pains and muscle weakness	^18^FDG-PET	surgical resection	pain free and returned to full activity	21 months	has no evidence of tumor recurrence
Sanjana Jayaraj Mangala et al. (20) (2025)	1	mandibular	elevated fibroblast growth factor-23; hypophosphataemia	bone pains and fracture	^68^Ga-DOTA-TATE-PET/CT	RFA (STARmed);	significant symptomatic improvement	No	NM
Yindong Liu et al. (21) (2025)	1	maxilla	low serum phosphorus; elevated alkaline phosphatase level; increased urinary phosphate	bone pains	^68^Ga-DOTA-TATE-PET/CT	argon-helium cryoablation	significant relief from weakness and pain	1.5 years	NM

## Discussion

We report a case of tumor-induced osteomalacia (TIO) secondary to a phosphaturic mesenchymal tumor of the jaw. The patient presented with generalized bone pain and bilateral lower limb weakness. Laboratory tests showed hypophosphatemia, and ^68^Ga-DOTA-TATE-PET/CT revealed a mass in the left mandible, raising the suspicion of hypophosphatemic osteomalacia. A mass was palpated in the jaw on physical examination, and further imaging assessed its location and relationship with surrounding tissues. Based on these clinical and laboratory findings, a diagnosis of TIO was made. The patient underwent surgical resection, after which serum phosphorus levels normalized and symptoms of bone pain and fatigue markedly improved.

TIO is a rare paraneoplastic syndrome characterized by renal phosphate wasting, resulting in hypophosphatemia and altered bone turnover (22). It is most frequently caused by PMTs, a rare and distinct group of neoplasms. Approximately 95% of PMTs involve the extremities and appendicular skeleton, whereas only 5% originate in the head and neck region, where involvement is uncommon and the nose and paranasal sinuses are predominantly affected (23). Clinically, patients present with bone pain, insufficiency fractures, and progressive muscular weakness. Biochemically, TIO is marked by hypophosphatemia and elevated alkaline phosphatase levels (24).

At present, most studies suggest that TIO-inducing tumors overexpress the protein FGF23, produced by small, slow-growing benign PMT (22). The protein FGF23 inhibits renal phosphate reabsorption in the proximal tubules, to cause hyperphosphaturia and refractory hypophosphatemia, eventually affecting bone mineralization (22). When PMTs induce FGF-23 production, which inhibits renal and intestinal phosphate absorption, chronic hypophosphatemia ensues. This persistent phosphate deficit impairs bone mineralization, ultimately leading to osteomalacia. FGFR1 is involved in the excessive induction of FGF-23 in inherited hypophosphatemic diseases. FN1-FGFR1 protein is thought to promote FGFR1 activation (25). Following tumor resection, serum FGF23 levels decline, serum phosphorus levels increase, and clinical symptoms improve markedly, with most patients achieving complete recovery. In light of the rarity of TIO, especially when localized to the jaw, we present a case of tumor-induced osteomalacia caused by a phosphaturic mesenchymal tumor of the jaw, accompanied by a review of the relevant literature.

Previous reports of jaw PMT-associated hypophosphatemic osteomalacia remain limited, with most cases described as isolated case reports. A review of the available literature indicates that PMTs arising in the jaw represent a rare subset of tumor-induced osteomalacia, accounting for only a small proportion of reported PMTs. The mandible appears to be more frequently involved than the maxilla; however, the clinical manifestations are highly variable. Similar to the present case, most reported patients developed systemic symptoms associated with chronic hypophosphatemia, including diffuse bone pain, muscle weakness, difficulty walking, and multiple fractures, while local jaw manifestations were often absent or nonspecific (3, 26).

Due to the rarity of jaw PMTs and their nonspecific clinical presentation, diagnosis is frequently delayed. Several reported cases were initially considered as odontogenic lesions, benign fibro-osseous lesions, or other maxillofacial tumors before the underlying metabolic abnormality was recognized. Biochemical evaluation, including serum phosphate, alkaline phosphatase, and renal phosphate wasting assessment, plays an essential role in establishing the diagnosis of tumor-induced osteomalacia. Regarding tumor localization, conventional imaging modalities may fail to identify small FGF23-producing tumors, whereas functional imaging targeting somatostatin receptors has demonstrated considerable value in patients with jaw PMTs. Previous cases have shown that ^68^Ga-DOTA-TATE-PET/CT can successfully localize SSTR-positive lesions and guide surgical management. Complete surgical excision remains the preferred treatment, with most reported patients achieving normalization of serum phosphate levels and significant improvement of osteomalacia-related symptoms after tumor removal (27, 28). Compared with previously reported jaw PMT cases, our patient presented with typical biochemical abnormalities of TIO and demonstrated successful localization of the mandibular lesion by ^68^Ga-DOTA-TATE-PET/CT, followed by complete surgical resection and rapid clinical improvement. These findings further emphasize the importance of considering jaw PMT in patients with unexplained hypophosphatemia and skeletal symptoms.

Although the lesion in this case was evident on physical examination, PMTs are often small and difficult to detect using conventional imaging modalities. In contrast, ^68^Ga-DOTA-TATE-PET/CT provides functional imaging based on somatostatin receptor expression, particularly SSTR2A, which is frequently overexpressed in PMTs. In the present case, ^68^Ga-DOTATATE PET/CT demonstrated focal radiotracer uptake in the left mandibular lesion, with a lesion SUVmax of 10.3, supporting the diagnosis of an SSTR-positive phosphaturic mesenchymal tumor and facilitating accurate preoperative localization. ^68^Ga-DOTA-TATE-PET/CT is a functional imaging modality used to localize tumors expressing somatostatin receptors (SSTR) (29). More recently, the PMTs has improved diagnostic imaging through the use of radiolabeled somatostatin (30). PMT is a highly vascularized tumor that frequently overexpresses the SSTR, mainly subtype 2A (25). Overexpression of SSTR is exploited by using radio-labelled somatostatin (SST) and its analogues, resulting in highly sensitive and specific imaging patterns. ^68^Ga-DOTA-TATE-PET/CT, is an antagonist of the SSTR, which upon receptor binding is internalized resulting in accumulation of radioactivity in tumor cells (25). ^68^Ga-DOTA-TATE-PET/CT is highly sensitive to identifying and quantifying PMTs. Previous studies have demonstrated that some mesenchymal tumors, particularly phosphaturic mesenchymal tumors (PMTs), exhibit high expression of SSTR2 on the cell membrane. Consequently, somatostatin analogues such as octreotide can specifically target SSTR2-expressing tumor cells for imaging and potential targeted therapy (25, 31).

In this case, the primary tumor (PMT) was initially undetected on conventional imaging. Subsequent ^68^Ga-DOTA-TATE-PET/CT revealed a somatostatin receptor-positive PMT localized in the left mandibular region. The presence of SSTR2-related mRNA in PMT tumor cells has been confirmed by RT-PCR technology, which provides a theoretical basis for the application of ^68^Ga-DOTA-TATE-PET/CT for the detection of PMT lesions, and a new treatment method for patients with hypophosphatemic osteomalacia caused by phosphaturic mesenchymal tumor (25). In the present case, ^18^F-FDG PET/CT failed to identify the causative lesion, whereas ^68^Ga-DOTATATE PET/CT demonstrated focal uptake in the left mandibular region. The different findings between ^18^F-FDG PET/CT and ^68^Ga-DOTATATE PET/CT may reflect the distinct biological targets of the two tracers. ^18^F-FDG uptake primarily reflects glucose metabolism, and small, slow-growing PMTs may exhibit relatively low FDG avidity, potentially limiting their detection. In contrast, ^68^Ga-DOTATATE targets somatostatin receptors, particularly SSTR2, which are frequently expressed in PMTs. Therefore, SSTR-targeted imaging may facilitate the localization of PMTs that are inconspicuous on ^18^F-FDG PET/CT.

TIO should be distinguished from hyperparathyroidism (HPT). Patients with hyperthyroidism may also have hypophosphatemic osteomalacia and systemic multiple fractures, because parathyroid hormone (PTH) promotes the renal tubule reabsorption of calcium ions and excretion of phosphate. Long-term excessive PTH secretion will stimulate osteoclast growth, the production of hypercalcium and hypophosphorus (32). As bone tissue is replaced by blood vessels and fibrous tissues in the middle of the repair process, myxosis and repeated bleeding may occur, and eventually form a soft tissue mass with uneven density, i.e., a mass comprised of granulation tissue containing multinucleated osteoclastic giant cells known as a brown tumor (33). Initially, osteoclastic multinucleated giant cells are abundant within the fibrovascular stroma of HPT brown tumors. Over time, fibro-osseous benign fibro osseous changes lacking giant cells may replace this stromal appearance. In HPT, there is generally low or normal serum phosphorus. Patients with severe hyperparathyroidism also have clinical manifestations of multiple systemic fractures and bone pain, which can be easily confused with PMT. Differential diagnosis can be made by an increased serum PTH and calcium ion concentration, as well as by using ultrasound or CT to explore for suspicious soft tissue nodules in the neck. In this case, both the PTH index and calcium ion concentration were normal, which can help rule out hypophosphatemic osteomalacia caused by hyperparathyroidism. ^68^Ga-DOTA-TATE-PET/CT can also be used to differentiate the two. Brown tumors may be misdiagnosed as tumors, however, as they do not represent SSTR2, ^68^Ga-DOTA-TATE-PET/CT should not be performed. Patients with chronic hepatitis B virus (HBV) generally prefer to be treated with adefovir dipivoxil (ADV), which can inhibit the synthesis of DNA in the mitochondria of the proximal tubular epithelial cells of the kidney, leading to cell necrosis, thereby reducing the kidney's reabsorption of phosphorus, resulting in hypophosphatemia and hyperphosphaturia with biochemical characteristics similar to those of PMT (34). Long-term, high-dose administration can also cause osteomalacia and muscle weakness. Therefore, when making a clinical diagnosis, it is necessary to consider the patient's history of long-term medication use.

Histologically, most PMTs are predominantly composed of abundant blood vessels and spindle-shaped or stellate mesenchymal cells. The tumors typically exhibit a relatively uniform appearance, characterized by small nuclei, inconspicuous nucleoli, abundant thick-walled and malformed vessels, and an absence of cytologic atypia. Pathological findings may include calcification and chondroid metaplasia. The spindle or stellate mesenchymal cells are embedded within a myxoid or myxochondroid matrix, which frequently demonstrates hemangioendothelioma-like vascular patterns. In addition, tumor cells produce a characteristic “smoky” matrix that, upon calcification, appears abnormally flocculent, villous, or dirty (31, 35). However, PMT remains relatively rare with heterogeneous pathological features, and standardized diagnostic criteria have yet to be established. Although the histopathological findings supported the diagnosis of PMT, immunohistochemical staining for PMT-associated markers, such as FGF23, SSTR2A, and CD56, was not performed in this case. Therefore, the lack of immunohistochemical confirmation should be acknowledged as a limitation of the pathological evaluation.

When PNS are suspected, these clinical signs should prompt further imaging investigations, particularly if the diagnosis remains unclear. Although PMT are rare, most are benign, and their key therapeutic hallmark is the reversal of biochemical/metabolic abnormalities post-resection. The generally accepted treatment principle is to first to treat the cause of the disease. Complete resection of the tumor has an efficacy rate of more than 90%. In most patients with this disease, the blood phosphorus level will return to normal in 1–2 weeks after surgery. Bone pain symptoms may be temporarily aggravated after surgery, but generally improve within 6 months. Drug treatment can also be used to supplement neutral phosphorus and calcitriol, active vitamin D, and calcium, and patients may need to take medication, several times a day for the rest of their lives to maintain effective blood drug concentration. Burosumab is a human monoclonal antibody against FGF-23, thus counteracting FGF-23 action. Burosumab normalizes phosphate levels if the tumor is not resectable or unlocalized, or when it recurs after surgery (36).

In patients with severe osteomalacia and extensive skeletal involvement, recovery of bone strength may lag behind biochemical correction. In such circumstances, anabolic agents such as teriparatide have been explored in other metabolic bone disorders to promote bone formation and accelerate skeletal recovery (37). However, evidence supporting their use in TIO remains limited, and correction of FGF23 excess through tumor removal remains the primary therapeutic approach.

Although an approximately 5-mm macroscopic surgical margin was achieved, a separate microscopic histopathological assessment of the resection margins was not documented. Therefore, continued clinical and biochemical follow-up is warranted to monitor for possible local recurrence. Although no tumor recurrence was observed in this case, the infiltrative nature of PMTs implies a non-negligible risk of local relapse. Blood phosphorus should thus be regularly reviewed after surgery, and tumor recurrence should be considered in cases of -recurring of blood phosphorus reduction, bone pain, or other related symptoms.

This rare case of TIO caused by PMTs highlight key clinical lessons. When patients present with unexplained bone pain or fractures, PMT-associated TIO should be considered, and comprehensive biochemical evaluation is warranted. Localization of the PMT is critical; if conventional imaging fails, ^68^Ga-DOTA-TATE-PET/CT should be employed. Upon identification, complete surgical resection is the definitive treatment, and removal of the tumour resolves the biochemical and metabolic abnormalities. Patients require follow-up for ≥6 months to monitor symptom resolution and bone recovery. In cases of recurrence, prompt reintervention is recommended.

## Data Availability

The original contributions presented in the study are included in the article/Supplementary Material, further inquiries can be directed to the corresponding authors.
